# Burden of *Clostridium (Clostridioides) difficile* Infection among Patients in Western Asia: A Systematic Review and Meta-Analysis

**Published:** 2019-09

**Authors:** Yalda MALEKZADEGAN, Mehrdad HALAJI, Meysam HASANNEJAD-BIBALAN, Saba JALALIFAR, Javad FATHI, Hadi Sedigh EBRAHIM-SARAIE

**Affiliations:** 1. Department of Bacteriology and Virology, School of Medicine, Shiraz University of Medical Sciences, Shiraz, Iran; 2. Department of Microbiology, School of Medicine, Isfahan University of Medical Sciences, Isfahan, Iran; 3. Poursina Clinical Research Development Unit, Guilan University of Medical Sciences, Rasht, Iran; 4. Department of Microbiology, School of Medicine, Guilan University of Medical Sciences, Rasht, Iran; 5. Razi Clinical Research Development Center, Guilan University of Medical Sciences, Rasht, Iran

**Keywords:** *Clostridium difficile* infection (CDI), Western Asia, Infection control, Meta-analysis

## Abstract

**Background:**

*Clostridium difficile* is the most common causes of hospital-acquired diarrhea affecting particularly hospitalized patients globally. This organism has re-emerged in recent years with significant morbidity and mortality. The present study aimed to estimate the burden of *C. difficile* infection (CDI) and to acquire information on the overall rates of community- and hospital-acquired CDI in western Asia.

**Methods:**

A systematic literature search was performed to identify articles published from the eight Persian Gulf countries in western Asia including Iran, Iraq, Bahrain, Kuwait, Oman, Qatar, Saudi Arabia, and the United Arab Emirates in the electronic databases within Jan of 2000 to Dec of 2017. Then, 20 publications which met our inclusion criteria were selected for data extraction and analysis by Comprehensive Meta-Analysis Software.

**Results:**

Twenty studies reported the prevalence of toxigenic strains of *C. difficile* among patients from Persian Gulf countries, of these the pooled prevalence of CDI was 9% (95% CI: 6.5%–12.5%). Totally, 8 studies showed the prevalence of hospital-acquired CDI, from those studies the prevalence of CDI was estimated 8.4% (95% CI: 4.9%–14.1%). Moreover, 7 studies reported the prevalence of community-acquired CDI, from those studies the prevalence of CDI was estimated 1.8% (95% CI: 1.2%–2.9%).

**Conclusion:**

The prevalence of CDI in western Asia is lower than southern and eastern region. Moreover, the lower prevalence of community-acquired CDI compared to hospital-acquired CDI, indicate that the source of infection in western Asia is more likely in the hospitals.

## Introduction

Hospital-acquired infections (HAIs) are a serious public health concern resulting in prolonged hospitalization and risk of death (1). The occurrence of HAIs according to the WHO estimates is around 7.1 million cases every year (2). Among the wide range of bacteria has been reported as a cause of HAIs, vancomycin-resistant enterococci (VRE), methicillin-resistant *Staphylococcus aureus* (MRSA), *Clostridium difficile*, *Acinetobacter baumannii*, *Pseudomonas aeruginosa*, and *Enterobacteriaceae* are the most prevalent pathogens (3, 4).

*Clostridium difficile* is the most common cause of hospital-acquired diarrhea affecting particularly hospitalized patients globally (5). This organism has re-emerged in recent years with apparent greater morbidity and mortality and also associated with increased health care costs (6). Factors contributing to the development of this phenomenon include usage of broad-spectrum antibiotics such as fluoroquinolones, clindamycin and third generation cephalosporin’s as well as prolonged hospitalization, antineoplastic chemotherapy, and severe underlying diseases (7, 8). Treatment of *C. difficile* infection (CDI) is challenging and demand new approaches because most of antibiotics used for the treatment of every kind of infections can potentially encourage CDI (9, 10). The currently available antibiotics that remain the first-line therapy for CDI are metronidazole and vancomycin (11, 12).

Antibiotic*-*associated diarrhea is mostly linked to strains of *C. difficile* that produce a range of virulence factors including toxins and adherence factors (13). Toxin A (TcdA) and toxin B (TcdB) are two homologous exotoxin and main virulence factors of toxigenic *C. difficile* that encoded by the *tcdA* and *tcdB* genes, respectively. Expression of these toxins causing proinflammatory and cytotoxic effects including disruption of the actin cytoskeleton and impairment of tight junctions in human intestinal epithelial cells that are responsible for the clinical symptoms of CDI (13).

The incidence of CDIs is an important quality indicator to reflect the effectiveness of the basic hospital policies include infection control and antimicrobial stewardship. The objectives of the present study were to estimate the burden of CDI and to acquire information on the overall rates of community- and hospital-acquired CDI in western Asia. Results from this survey indicate progress across Persian Gulf countries towards comprehensive monitoring and reporting of CDI.

## Methods

### Search strategies

A systematic literature search was performed to identify papers published from the eight Persian Gulf countries in western Asia including Iran, Iraq, Bahrain, Kuwait, Oman, Qatar, Saudi Arabia, and the United Arab Emirates in the Web of Science, PubMed, Scopus, and Google Scholar electronic databases within Jan of 2000 to Dec of 2017. The keywords and terms were searched using Medical Subject Headings (MeSH) such as “*Clostridium difficile*” or “*Clostridioides difficile*” or “*C. difficile*” or “*Clostridium difficile* infection (CDI)” or “Pseudomembranous colitis” in combination with “Names of countries” in the title, abstract and keywords fields.

### Selection criteria

Two reviewers independently screened the search results at the databases with the related keywords and analysis the titles, abstracts, and full texts to applied eligibility for inclusion according to inclusion criteria, and the disagreement between reviewers was resolved by consensus. English and Persian or Arabian language articles with English abstract indexed in PubMed, Scopus or Web of Science with the following criteria were considered in our study: 1) Clearly mentioned to method used for *C. difficile* and toxins detection; 2) cross-sectional or retrospective studies investigating the prevalence of toxigenic *C. difficile* collected from diarrhea samples. Meanwhile, exclusion criteria were: 1) studies that did not report a standardized method for detection of *C. difficile* and toxins; 2) studies with sample size was less than 10 isolates; 3) studies that origin of samples was unclear or isolates obtained from formed stool or environment sources, and 4) studies which focused on non-toxigenic *C. difficile* or the prevalence of toxigenic strains was unclear. Furthermore, reviews and systematic review articles, case reports, and articles which were only available in abstract form were ignored.

### Definition

According to the European Center for Disease Prevention and Control (ECDC), an episode of CDI was defined as a patient with diarrhea whose stool takes the shape of the container, and it is positive for *C. difficile* toxin A and/ or B without other etiology (14).

### Quality assessment

The quality of eligible studies was judged independently by two authors using the STROBE checklist (Strengthening the Reporting of Observational Studies in Epidemiology). Items related title and abstract, introduction, methods, results, discussion, and other information were determined and a score was assigned to each item. One score was assigned to each question and studies achieved at least eight quality scores were considered eligible and included in the study (15).

### Data extraction

For all selected studies, the following details were extracted: the first author’s name, the study performing time, publication date, research location, sample type, patients age range, nature of patients (hospitalized or outpatient), toxins detection methods, primary sample size, the frequency of toxigenic *C. difficile*, source of infection, origin of infection, and proportion of toxigenic strains in each gender.

### Statistical analysis

Analysis of data was performed using Comprehensive Meta-Analysis Software Ver. 2.2 (BioStat Company). Meta-analysis was performed using random-effects model to estimate the pooled prevalence and corresponding 95% confidence interval (CI). Statistical heterogeneity between and within groups was determined using Cochran’s Q statistic and the I^2^ index. The funnel plot, Begg’s rank correlation test, and Egger’s weighted regression tests were used to evaluate possible publication bias (*P*<0.05 is indicative of publication bias).

The present study designed according to the Preferred Reporting Items for Systematic Reviews and Meta-Analyses (PRISMA) guidelines.

## Results

**Fig. 1:**
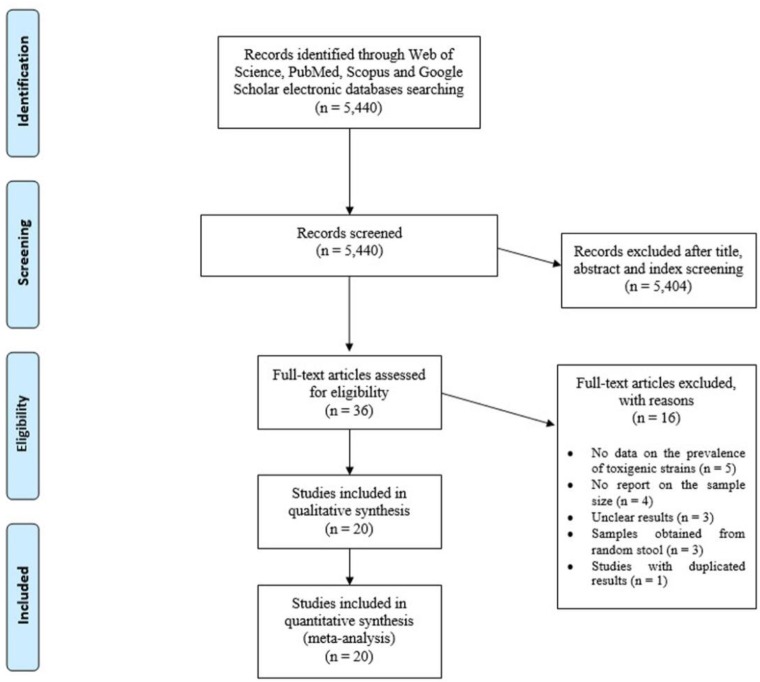
Flow chart of the literature search strategy and study selection

Initially, 5440 citations were yielded from the database search. Among them, 5404 were excluded on the initial screening of the index, title and abstract and 36 were reviewed in full text. Of 36 full text reviewed articles, five studies did not report the prevalence of toxigenic strains of *C. difficile*, four studies did not report primary samples size, three studies had unclear results, three studies performed on random stools, and results of one study duplicated in their recent study. Finally, 20 studies were eligible for inclusion and were subjected to meta-analysis. A flowchart of the literature search, the selection procedures and reasons for exclusion are presented in Fig. 1. The characteristics of the included studies in the meta-analysis are available in Table 1.

**Table 1:** Characteristics of studies included in the meta-analysis

***First author***	***Publication year***	***Preformed time***	***Country***	***Sample type***	***Age range***	***Hospitalized (H) or outpatient (OP)***	***Detection method***	***Sample size***	***Toxigenic C. difficile No.***	***HA/CA No.***	***M/F No.***	***Ref***
Sadeghifard	2010	2002–2006	Iran	Diarrheal stool	No limit	H	Cytotoxicity assay	942	57	-	30/27	(16)
Nazemalhosseini-Mojarad	2011	UN	Iran	Diarrheal stool	No limit	H and OP	ELISA	356	19	-	13/6	(17)
Nasri	2012	2009–2010	Iran	Diarrheal stool	No limit	H	ELISA	162	36	-	24/12	(18)
Jalali	2012	2010–2011	Iran	Diarrheal stool	No limit	H	Molecular	86	17	13/4	9/8	(19)
Goudarzi	2013	2010–2011	Iran	Diarrheal stool	No limit	H	Molecular	350	75	-	39/36	(20)
Farshad	2013	2012	Iran	Nosocomial diarrhea	No limit	H	Cytotoxicity assay and ELISA	122	9	9/-	5/4	(21)
Azizi	2013	2010	Iran	Nosocomial diarrhea	No limit	H	Molecular	98	15	-	-	(22)
Alinejad	2015	2013–2014	Iran	Nosocomial diarrhea	6–60 months	H	ELISA	38	8	8/-	6/2	(23)
Rezazadeh Zarandi	2017	2014–2015	Iran	Diarrheal stool	No limit	H	Molecular and ELISA	233	11	-	-	(24)
Azimirad	2017	2011–2012	Iran	Diarrheal stool	No limit	H	Molecular	105	18	-	9/10	(25)
Sandokji	2009	2007–2008	Saudi Arabia	Diarrheal stool	>2 yr	H	ELISA	258	56	56/-	-	(26)
Al-Tawfiq	2010	2007–2008	Saudi Arabia	Diarrheal stool	No limit	H and OP	ELISA	913	42	26/16	23/19	(27)
AL-Eidan	2013	2011	Saudi Arabia	Diarrheal stool	Adult	H	ELISA	2927	171	98/73	-	(28)
Senok	2017	2014–2015	Saudi Arabia	Diarrheal stool	No limit	H and OP	Molecular	210	31	-	-	(29)
Jamal	2010	2003–2005	Kuwait	Diarrheal stool	No limit	H and OP	ELISA	697	73	56/17	-	(30)
Jamal	2014	2012	Kuwait	Diarrheal stool	>2 yr	OP	Molecular	409	13	-/13	-	(31)
Jamal	2015	2011–2013	Kuwait	Diarrheal stool	>2 yr	OP	ELISA	2548	16	-/16	-	(32)
Albert	2016	2014–2015	Kuwait	Diarrheal stool	No limit	H	Molecular	109	3	-	-	(33)
Alrifai	2009	2004–2005	Iraq	Nosocomial diarrhea	1–60 months	H	ELISA	81	17	-		(34)
Al-Thani	2014	2011–2012	Qatar	Diarrheal stool	>1 yr	H and OP	EIA and Molecular	1,532	122	98/14	72/50	(35)

Abbreviations: ELISA: enzyme-linked immunosorbent assay; EIA: Enzyme Immunoassay; HA: hospital-acquired; CA: community-acquired; M: male; F: female.

Of the total number of included studies, 10 of which were from Iran (16–25), four from Saudi Arabia (26–29), four from Kuwait (30–33), and one for each of Iraq (34), and Qatar countries (35). Totally, 13 studies conducted only among hospitalized patients, five studies contained both hospitalized and outpatients and two studies enrolled only outpatients.

**Fig. 2:**
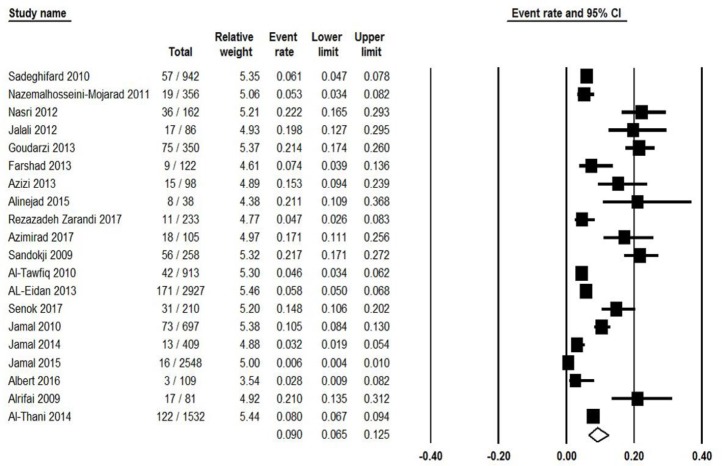
Forest plot of the pooled prevalence of CDI in western Asia

**Fig. 3:**
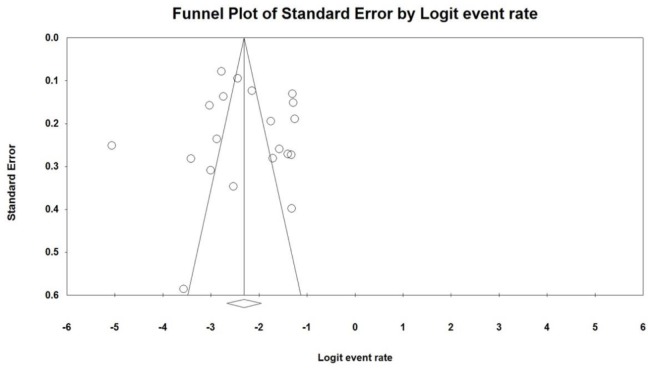
Funnel plot of meta-analysis on CDI in western Asia

Twenty studies reported the prevalence of toxigenic strains of *C. difficile* among patients from Persian Gulf countries, of these the pooled prevalence of CDI was 9% (95% CI: 6.5%–12.5%) ranging from 0.6% to 22.2% (Fig. 2). There was a significant heterogeneity among the included studies (χ^2^ = 410.893; *P*<0.001; I^2^ =95.4%). Moreover, a symmetric funnel plot of the included studies showed no evidence of publication bias (Fig. 3). Additionally, Begg’s and Egger’s tests were performed to quantitatively evaluate the probable publication bias among studies. According to the results of Begg’s test (z=0.097, *P*=0.92) and Egger’s test (t=0.34, *P*=0.74), no evidence of publication bias was observed.

**Fig. 4:**
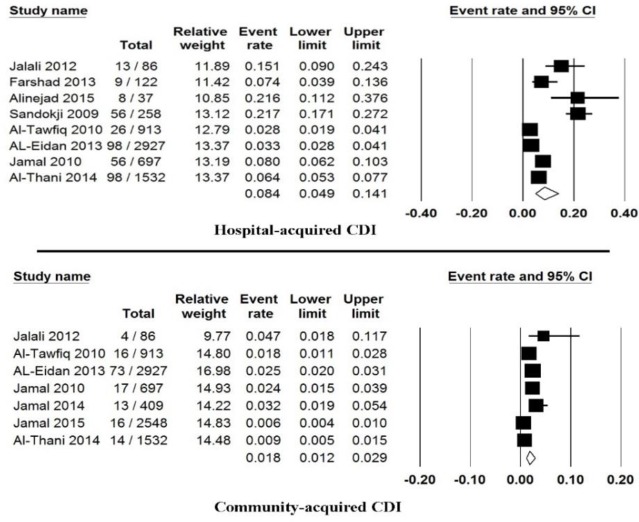
Forest plot of the pooled prevalence of community- or hospital-acquired

Totally, eight studies showed the prevalence of hospital-acquired CDI, from those studies the prevalence of CDI was estimated 8.4% (95% CI: 4.9%–14.1%) ranging from 2.8% to 21.7% (Fig. 4).

**Fig. 5:**
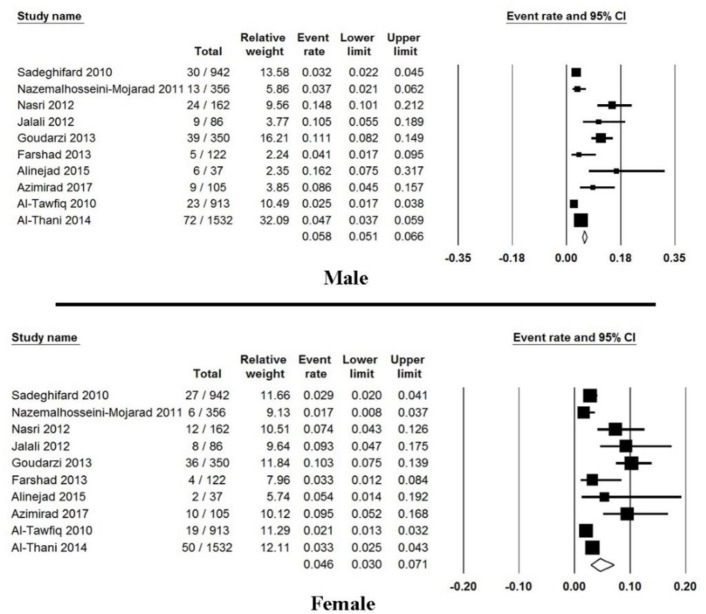
Forest plot of the pooled prevalence of male and female patients with CDI

**Fig. 6:**
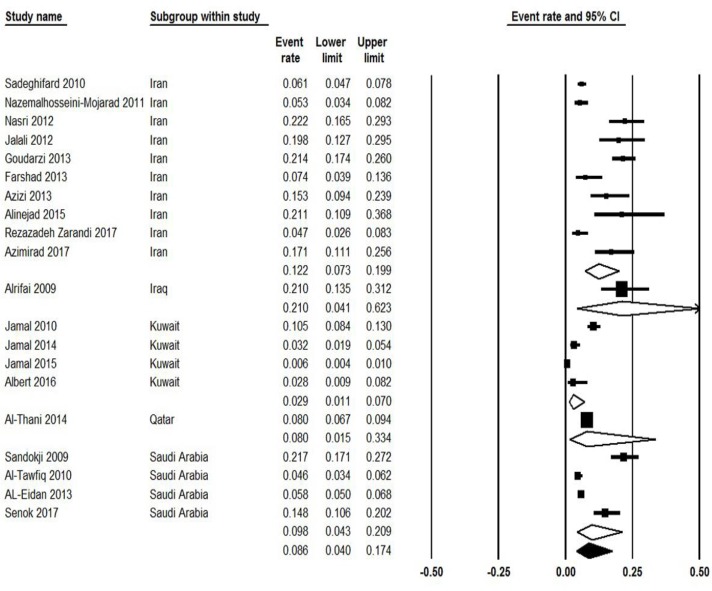
Forest plot of subgroups analysis of the pooled prevalence of CDI between countries in western Asia

Moreover, seven studies reported the prevalence of community-acquired CDI, from those studies the prevalence of CDI was estimated 1.8% (95% CI: 1.2%–2.9%) ranging from 0.6% to 4.7% (Fig. 4). Ten studies showed the incidence of CDI regarding gender, from those studies the occurrence of CDI among male and female patients was estimated 6% (95% CI: 4%–9%) and 4.6% (95% CI: 3.0%–7.1%), respectively (Fig. 5). Finally, subgroups analysis between countries were done and results were presented in Fig. 6.

## Discussion

The emergence of the hypervirulent *C. difficile* strains increase the prevalence and severity of CDI; however, epidemic strain of NAP1/BI/027 is not common in our region and is a public health problem for other countries, mainly western countries (36). Thus, it is essential to gain a close estimation of the burden of CDI for the development of effective healthcare practice. To the best of our knowledge, the present study is the largest comprehensive survey to date estimated the pooled prevalence of CDI 9% from the Persian Gulf region. Moreover, the prevalence of CDI differed greatly between studied countries and even hospitals in the same country ranging from 0.6% to 22.2%. Despite the discrepancy in literature, our findings are consistent with the median values reported in previous studies. In this regard, the prevalence of CDI in South and East Asia were reported 10.9% in India (37), 14% in China (38), and 14.3% in South-Korea (39). These reports, accompanied by results from a meta-analysis study that indicated the prevalence of CDI in Eastern Asia is higher than other parts (40). In American countries, this rate was reported 8% in Brazil (41), and 13.7% in the USA (42). While a hospital-based survey within 34 European countries showed much more discrepancy in the prevalence of CDI ranging from 0% in Luxembourg to 39% in Poland (43). These variations might be due to differences in predominant epidemic strains, geographical distribution, studied population or the sensitivity of detection methods.

In the present study, from those studies that reported the origin of infection, the prevalence of hospital-acquired CDI was estimated at 8.4%, while the prevalence of community-acquired CDI was 1.8%. International estimation of the burden of community- or hospital-acquired CDI is challenging since in most of the studies the origin of infection was not investigated. Comparison with available reports indicate that the prevalence of community-acquired CDI in our study (1.8%) is closest to reports from Brazil (1%) (41), South-Korea (1.6%) (39), and Europe (∼2%) (43), whereas it is lower than report from China (8%) (44).

The strengths of this systematic review based study were the large number of patients from the major countries of Middle East with a study design adherence to international guidelines for the estimating burden of CDI. Based on the ECDC experiences, continuous or periodical surveillance supplemented with epidemiological and microbiological data is a practical strategy for monitoring CDI (45). Meanwhile, increasing the national coverage of hospital-based CDI surveillance will improve the national estimates of the CDI burden (43, 45).

As the main limitation of the present study, our report was not representative of the actual rate in the Persian Gulf region. Since our estimation was based on published data, so the burden of infection would be expected to be different if a network of data from more hospitals and laboratories in this region were available. Moreover, differences in the severity of illness of patients or antibiotics prescription might be an effect on reporting rates (43).

## Conclusion

The results of the present study provide good epidemiological information about the distribution of CDI in the Persian Gulf region in western Asia. The prevalence of CDI in western Asia is lower than southern and eastern region. Moreover, the lower prevalence of community-acquired CDI compared to hospital-acquired CDI, indicate that the source of infection in western Asia is more likely in the hospitals. These findings highlighting the importance of active surveillance, and the demand for improving infection control policy in reducing the risk of CDI in hospitals.

## Ethical consideration

Ethical issues (Including plagiarism, informed consent, misconduct, data fabrication and/or falsification, double publication and/or submission, redundancy, etc.) have been completely observed by the authors.

## References

[1] Revelas A (2012). Healthcare-associated infections: A public health problem. Niger Med J, 53(2):59–64. 23271847 10.4103/0300-1652.103543 PMC3530249

[2] Sarani H Balouchi A Masinaeinezhad N Ebrahimitabas E (2015). Knowledge, Attitude and Practice of Nurses about Standard Precautions for Hospital-Acquired Infection in Teaching Hospitals Affiliated to Zabol University of Medical Sciences (2014). Glob J Health Sci, 8(3):193–8. 26493432 10.5539/gjhs.v8n3p193 PMC4804055

[3] Dancer SJ (2014). Controlling hospital-acquired infection: focus on the role of the environment and new technologies for decontamination. Clin Microbiol Rev, 27(4):665–90. 25278571 10.1128/CMR.00020-14 PMC4187643

[4] Asadian M Sadeghi J Rastegar Lari A (2016). Antimicrobial resistance pattern and genetic correlation in Enterococcus faecium isolated from healthy volunteers. Microb Pathog, 92:54–9. 26747584 10.1016/j.micpath.2015.12.014

[5] Heinlen L Ballard JD (2010). Clostridium difficile infection. Am J Med Sci, 340(3):247–52. 20697257 10.1097/MAJ.0b013e3181e939d8 PMC2935936

[6] Depestel DD Aronoff DM (2013). Epidemiology of Clostridium difficile infection. J Pharm Pract, 26(5):464–75. 24064435 10.1177/0897190013499521 PMC4128635

[7] Bignardi GE (1998). Risk factors for Clostridium difficile infection. J Hosp Infect, 40(1):1–15. 9777516 10.1016/s0195-6701(98)90019-6

[8] Surawicz CM (2015). Clostridium difficile infection: risk factors, diagnosis and management. Curr Treat Options Gastroenterol, 13(1):121–9. 25567105 10.1007/s11938-014-0038-3

[9] Sedigh Ebrahim-Saraie H Heidari H Amanati A (2018). A multicenter-based study on epidemiology, antibiotic susceptibility and risk factors of toxigenic Clostridium difficile in hospitalized patients in southwestern Iran. Infez Med, 26(4):308–15. 30555133

[10] Khan FY Abu-Khattab M Anand D (2012). Epidemiological features of Clostridium difficile infection among inpatients at Hamad General Hospital in the state of Qatar, 2006–2009. Travel Med Infect Dis, 10(4):179–85. 22800937 10.1016/j.tmaid.2012.06.004

[11] Nasiri MJ Goudarzi M Hajikhani B (2018). Clostridioides (Clostridium) difficile infection in hospitalized patients with antibiotic-associated diarrhea: A systematic review and meta-analysis. Anaerobe, 50:32–7. 29408016 10.1016/j.anaerobe.2018.01.011

[12] Di X Bai N Zhang X (2015). A meta-analysis of metronidazole and vancomycin for the treatment of Clostridium difficile infection, stratified by disease severity. Braz J Infect Dis, 19(4):339–49. 26001980 10.1016/j.bjid.2015.03.006 PMC9427463

[13] Di Bella S Ascenzi P Siarakas S (2016). Clostridium difficile Toxins A and B: Insights into Pathogenic Properties and Extraintestinal Effects. Toxins (Basel), 8(5):E134. 27153087 10.3390/toxins8050134 PMC4885049

[14] Jamal WY Rotimi VO (2016). Surveillance of Antibiotic Resistance among Hospital- and Community-Acquired Toxigenic Clostridium difficile Isolates over 5-Year Period in Kuwait. PLoS One, 11(8):e0161411. 27536994 10.1371/journal.pone.0161411 PMC4990247

[15] Bialvaei AZ Kouhsari E Salehi-Abargouei A (2017). Epidemiology of multidrug-resistant Acinetobacter baumannii strains in Iran: a systematic review and meta-analysis. J Chemother, 29(6):327–37. 28622734 10.1080/1120009X.2017.1338377

[16] Sadeghifard N Salari MH Ghassemi MR (2010). The incidence of nosocomial toxigenic clostridium difficile associated diarrhea in Tehran tertiary medical centers. Acta Med Iran, 48(5):320–5. 21287466

[17] Nazemalhosseini-Mojarad E Azimirad M Razaghi M (2011). Frequency of Clostridium difficile among patients with gastrointestinal complaints. Gastroenterol Hepatol Bed Bench, 4(4):210–3. 24834184 PMC4017434

[18] Nasri MR Khorvash F Zolfaghari MR Mobasherizadeh S (2012). The relative frequency of clostridium difficile in fecal samples of hospitalized patients with diarrhea by elisa method. J Isfahan Med School, 29(167):2376–82.

[19] Jalali M Khorvash F Warriner K Weese JS (2012). Clostridium difficile infection in an Iranian hospital. BMC Res Notes, 5:159. 22436392 10.1186/1756-0500-5-159 PMC3317812

[20] Goudarzi M Goudarzi H Alebouyeh M (2013). Antimicrobial susceptibility of clostridium difficile clinical isolates in iran. Iran Red Crescent Med J, 15(8):704–11. 24578839 10.5812/ircmj.5189 PMC3918196

[21] Farshad S Azami M Pouladfar G (2013). Prevalence and risk factors of Clostridium difficile-Associated diarrhea in Iranian hospitalized patients. Ann Trop Med Public Health 6(5):554–8.

[22] Azizi O Aslani MM Azimi Rad M (2013). The frequency of toxigenic strains of Clostridium difficile in hospitalized patients with diarrhea in Tehran/Iran by PCR method, 2010. J Kerman Univ Med Sci, 20(2):129–37.

[23] Alinejad F Barati M Satarzadeh Tabrisi M Saberi M (2015). Hospital acquired diarrhea in a burn center of Tehran. Iran J Microbiol, 7(6):310–4. 26885330 PMC4752684

[24] Rezazadeh Zarandi E Mansouri S Nakhaee N (2017). Frequency of antibiotic associated diarrhea caused by Clostridium difficile among hospitalized patients in intensive care unit, Kerman, Iran. Gastroenterol Hepatol Bed Bench, 10(3):229–34. 29118940 PMC5660274

[25] Azimirad M Krutova M Nyc O (2017). Molecular typing of Clostridium difficile isolates cultured from patient stool samples and gastroenterological medical devices in a single Iranian hospital. Anaerobe, 47:125–8. 28501554 10.1016/j.anaerobe.2017.05.004

[26] Sandokji AM Murshid KR El-Badry AA (2009). Infectious nosocomial diarrhea in the surgical wards: Role of parasites and microbes imply stool analysis. J Taibah Univ Med Sci, 4(1):73–81.

[27] Al-Tawfiq JA Abed MS (2010). Clostridium difficile-associated disease among patients in Dhahran, Saudi Arabia. Travel Med Infect Dis, 8(6):373–6. 21030314 10.1016/j.tmaid.2010.10.003

[28] Al-Eidan FA (2013). Proton pump inhibitors and the increased risk of Clostridium difficile infections: A case-control study. Int J Pharma Bio Sci, 4(2):B735–41.

[29] Senok AC Aldosari KM Alowaisheq RA (2017). Detection of clostridium difficile antigen and toxin in stool specimens: Comparison of the C. difficile quik chek complete enzyme immunoassay and GeneXpert C. difficile polymerase chain reaction assay. Saudi J Gastroenterol, 23(4):259–62. 28721981 10.4103/sjg.SJG_80_17 PMC5539681

[30] Jamal W Rotimi VO Brazier J Duerden BI (2010). Analysis of prevalence, risk factors and molecular epidemiology of Clostridium difficile infection in Kuwait over a 3-year period. Anaerobe, 16(6):560–5. 20887795 10.1016/j.anaerobe.2010.09.003

[31] Jamal W Pauline EM Rotimi VO (2014). Comparative performance of the GeneXpert C. difficile PCR assay and C. diff Quik Chek Complete kit assay for detection of Clostridium difficile antigen and toxins in symptomatic community-onset infections. Int J Infect Dis, 29:244–8. 25462186 10.1016/j.ijid.2014.10.025

[32] Jamal W Pauline E Rotimi V (2015). A prospective study of community-associated Clostridium difficile infection in Kuwait: Epidemiology and ribotypes. Anaerobe, 35(Pt B):28–32. 10.1016/j.anaerobe.2015.06.006 26144314

[33] Albert MJ Rotimi VO Iqbal J Chehadeh W (2016). Evaluation of the xTAG Gastrointestinal Pathogen Panel Assay for the Detection of Enteric Pathogens in Kuwait. Med Princ Pract, 25(5):472–6. 27322647 10.1159/000447698 PMC5588498

[34] Alrifai SB Alsaadi A Mahmood YA (2009). Prevalence and etiology of nosocomial diarrhoea in children < 5 years in Tikrit teaching hospital. East Mediterr Health J, 15(5):1111–8. 20214124

[35] Al-Thani AA Hamdi WS Al-Ansari NA (2014). Polymerase chain reaction ribotyping of Clostridium difficile isolates in Qatar: a hospital-based study. BMC Infect Dis, 14:502. 25223337 10.1186/1471-2334-14-502 PMC4262129

[36] Quesada-Gomez C Lopez-Urena D Acuna-Amador L (2015). Emergence of an outbreak-associated Clostridium difficile variant with increased virulence. J Clin Microbiol, 53(4):1216–26. 25653402 10.1128/JCM.03058-14 PMC4365207

[37] Vaishnavi C Singh M Mahmood S Kochhar R (2015). Prevalence and molecular types of Clostridium difficile isolates from faecal specimens of patients in a tertiary care centre. J Med Microbiol, 64(11):1297–304. 26361995 10.1099/jmm.0.000169

[38] Tang C Cui L Xu Y (2016). The incidence and drug resistance of Clostridium difficile infection in Mainland China: a systematic review and meta-analysis. Sci Rep, 6:37865. 27897206 10.1038/srep37865 PMC5126672

[39] Kwon SS Gim JL Kim MS (2017). Clinical and molecular characteristics of community-acquired Clostridium difficile infections in comparison with those of hospital-acquired *C. difficile*. Anaerobe, 48:42–6. 28655581 10.1016/j.anaerobe.2017.06.014

[40] Borren NZ Ghadermarzi S Hutfless S Ananthakrishnan AN (2017). The emergence of Clostridium difficile infection in Asia: A systematic review and meta-analysis of incidence and impact. PLoS One, 12(5):e0176797. 28463987 10.1371/journal.pone.0176797 PMC5413003

[41] Pires RN Monteiro AA Carneiro LC (2014). Clostridium difficile infection in Brazil: a neglected problem? Am J Infect Control, 42(4):459–60. 24559593 10.1016/j.ajic.2013.10.012

[42] Kilic A Alam MJ Tisdel NL (2015). Multiplex Real-Time PCR Method for Simultaneous Identification and Toxigenic Type Characterization of Clostridium difficile From Stool Samples. Ann Lab Med, 35(3):306–13. 25932438 10.3343/alm.2015.35.3.306 PMC4390698

[43] Bauer MP Notermans DW van Benthem BH (2011). Clostridium difficile infection in Europe: a hospital-based survey. Lancet, 377(9759):63–73. 21084111 10.1016/S0140-6736(10)61266-4

[44] Zhang D Chen J Zhan H (2016). Clostridium difficile-associated clinical burden from lack of diagnostic testing in a Chinese tertiary hospital. J Hosp Infect:S0195-6701(16)30435-2. 10.1016/j.jhin.2016.10.001 28029470

[45] van Dorp SM Kinross P Gastmeier P (2016). Standardised surveillance of Clostridium difficile infection in European acute care hospitals: a pilot study, 2013. Euro Surveill, 21(29): 10.2807/1560-7917.ES.2016.21.29.30293. 10.2807/1560-7917.ES.2016.21.29.30293 27472820

